# New statistical method identifies cytokines that distinguish stool microbiomes

**DOI:** 10.1038/s41598-019-56397-9

**Published:** 2019-12-27

**Authors:** Dake Yang, Jethro Johnson, Xin Zhou, Elena Deych, Berkley Shands, Blake Hanson, Erica Sodergren, George Weinstock, William D. Shannon

**Affiliations:** 1 BioRankings, St. Louis, MO USA; 2 Jackson Laboratory for Genomic Medicine, Hartford, CT USA; 3 University of Texas Health Sciences Center, Houston, TX USA; 4 Washington University School of Medicine, St Louis, MO USA

**Keywords:** Bioinformatics, Bacterial genetics

## Abstract

Regressing an outcome or dependent variable onto a set of input or independent variables allows the analyst to measure associations between the two so that changes in the outcome can be described by and predicted by changes in the inputs. While there are many ways of doing this in classical statistics, where the dependent variable has certain properties (e.g., a scalar, survival time, count), little progress on regression where the dependent variable are microbiome taxa counts has been made that do not impose extremely strict conditions on the data. In this paper, we propose and apply a new regression model combining the Dirichlet-multinomial distribution with recursive partitioning providing a fully non-parametric regression model. This model, called DM-RPart, is applied to cytokine data and microbiome taxa count data and is applicable to any microbiome taxa count/metadata, is automatically fit, and intuitively interpretable. This is a model which can be applied to any microbiome or other compositional data and software (R package *HMP*) available through the R CRAN website.

## Introduction

Researchers may want to know how microbiome composition (outcome or dependent variable) changes in relation to patient characteristics (inputs or independent variables) such as age, disease severity, or cytokine level. The value of this type of analysis is to understand how patient characteristics may directly impact microbial compositional and variability. For example, if we know the microbiome changes in a systematic way as patients age, those changes due to age can be accounted for and removed when studying disease processes and the differences between healthy and sick patients^1–3^. If the composition of the taxa population is different for changing levels of cytokines, these can be examined as a possible mechanism of disease or for treatment targets^4^.

Associating patient outcomes (dependent variable) with input covariates (independent variables) is often a regression analysis problem to statisticians. Regression methods from classical statistics have been developed such as linear regression for numerical outcomes like cholesterol levels, logistic regression for binary outcomes like responder versus non-responder, or proportional hazards regression for survival analyses like time to death^5^. As computing power increased over the years, new regression algorithms have emerged that apply to more complex models and outcomes. These include nonlinear, mixed, and nonparametric regression (e.g., splines) models^6^, and nonparametric recursive partitioning to finds regions of the covariate space where the outcome is more homogeneous^7,8^. Within the recursive partitioning framework even more complex outcomes have been modeled such as survival models^9^ or Haseman-Elston models from genetic epidemiology to associate genetic markers in sibpairs with disease^10^.

However, these models won’t work off-the-shelf when the outcome (dependent variable) are the taxa counts and the inputs (independent variables) are the metadata since the mathematics and computing behind them don’t apply to microbiome taxa count outcomes. To get around this, researchers have developed *ad hoc* regression methods. For example, we regressed individual taxa proportions (Bacilli, Clostridia, and Gammaproteobacteria) onto day of life, days on antibiotics, breast milk, and C-section in a study of necrotizing enterocolitis in pre-term births to see how these variables impacted the levels of these taxa^11^. Since each taxon was regressed separately, this approach was suboptimal by not using the covariation information among the taxa and metadata in the analysis, and it was not possible to model how all three taxa changed simultaneously. A second approach uses random forests, however these models use the individual taxa as inputs or independent variables to predict an outcome or dependent variable, such as a source of pollution^12^. While this is an important question, it is addressing the inverse problem of how does the microbiome composition change as vales of different meta-data (inputs) change. It also treats each taxon separately ignoring the information contained in the covariance structure of the taxa. A third approach is to cluster samples using distance measures calculated from the microbiome data and compare the metadata distributions across subgroups, such as done to detect differences in microbiome composition between non-demented and demented patients^13^. The weakness of this approach is that the clustering and analysis metadata are independent and not using the joint information inherent in the multivariate taxa count/metadata data resulting in a less powerful statistical analysis.

In regression the analysis of microbiome compositions and metadata should be done jointly to make full use of the covariance information in the data. Further, we are interested in ‘regressing microbiome data onto metadata’ which identifies the taxa counts as the outcome or dependent variable, and the metadata as the input or independent data. One attempt to regress microbiome data onto metadata has been previously proposed^14^. However, the sparseness of the taxa count data makes estimating regression coefficients and error terms difficult, and often these cannot be estimated.

**Figure 1:**
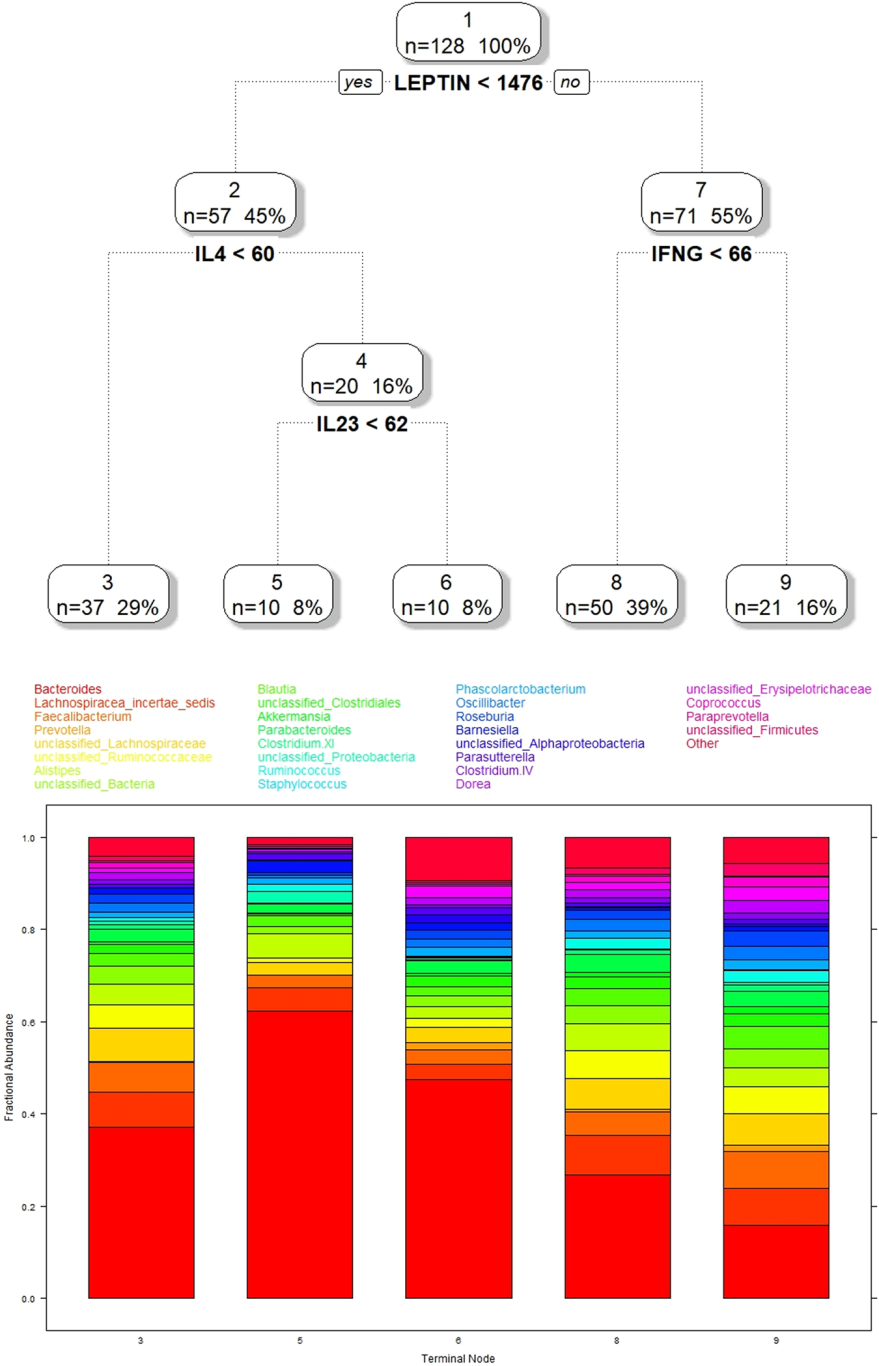
Results of applying DM-RPart to the iHMP microbiome and cytokine data. 1(**A**) (top) shows the optimal tree found by fitting the full tree and using cross-validation pruning to find the best fitting tree. 1(**B**) (bottom) shows the taxa frequency composition differences for the 5 terminal nodes.

Regression, when the outcomes are microbiome taxa counts or compositions, will allow investigators to predict how taxa counts change for different values of covariates. In this paper we propose a novel and practical approach for regressing microbiome data onto metadata that analyzes the data jointly, is automatic and computationally easy, and is intuitively interpretable. This method combines the Dirichlet-multinomial probability model which has been shown to apply to microbiome compositional data^15^, and the well-established nonparametric recursive partitioning regression framework^16^. The Dirichlet-multinomial recursive partitioning model, DM-RPart, takes the taxa compositions as the output or dependent variable, and the metadata as the inputs and finds partitions of the metadata where the microbiome samples differ. The following sections describe the algorithm and give an example showing how different cytokine values result in different microbiome composition. DM-RPart is available in the R HMP package as open source code through CRAN. In addition, the data and code to generate Fig. 1 are included as a vignette and can be used to reproduce our results and as a template for running other datasets.

## Regression With Microbiome Composition as The Outcome

The method presented here has been developed for microbiome compositional data outcomes using recursive partitioning that separates samples into non-overlapping subgroups based on patient covariate values – samples within a subgroup are more similar to each other in their microbial composition than to samples in other subgroups. Similarity of a separation into two subgroups is measured by the Dirichlet-multinomial (DM) model loglikelihood ratio^15,17^. The Dirichlet-multinomial recursive partitioning algorithm (DM-RPart) allows the regression of the compositional data onto the covariates without having to resort to one-taxa-at-a-time, complex assumptions, or data reduction.

### Dm-rpart application to ihmp data

To demonstrate the utility of DM-RPart for investigating host covariate—microbiome interactions we curated a longitudinal dataset generated as part of the iHMP to test the hypothesis that changes in cytokines representing one or more physiological processes produce changes in the composition of the gut microbiome. These data consist of host-serum cytokines and gut microbiome samples collected from individuals during periods of self-reported viral upper respiratory infection. This work was supported by the NIH Common Fund Human Microbiome Project (HMP) 164(1U54DE02378901)^18,19^. Twelve cytokines were selected that may represent one or more physiological process associated with antimicrobial activity (IL-17F, IL-17A, IL-21, IL-22, IL-23, IL-12p40)^20^, autoimmunity (Eotaxin)^21^, allergy (IL-4, IL-13)^22^, and viral infection (IFNG)^23^, as well as cytokines related to obesity (Leptin)^24^ and regulation of inflammation (TGFB)^25^. Partitioning all 128 microbiome samples produced an optimal tree that first split samples based on leptin, then partitioned high-leptin microbiomes based on IFNG, and low-leptin microbiomes based on IL4/IL23 (Fig. 1A). Specifically, the DM-RP model partitions the 128 samples into 5 non-overlapping groups: 37 samples with Leptin < 1476 and IL4 < 60 (Terminal Node 3); 10 samples with Leptin < 1476, IL4 > 60, and IL23 < 62 (Terminal Node 5); 10 samples with Leptin < 1476, IL4 > 60, IL-23 > 62 (Terminal Node 6), 50 samples with Leptin > = 1476 AND IFNG < 66 (Terminal Node 8); and 21 samples with Leptin >  = 1476 AND IFNG > = 66 (Terminal Node 9). The percentages in each node represent the percent of all 128 samples. The terminal nodes at the bottom of the tree represent the subgroups. Selection of the variables and cut points is an automatic process based on optimizing a measure of *homogeneity* such that the samples in two child nodes, say the 57 samples with Leptin < 1476 and the 71 samples with Leptin >1476 are more homogeneous compared to when these samples are combined in the parent node above them. Figure 1B shows the composition of different genera in the terminal nodes, or subgroups, in Fig. 1. Note that DM-RP is data-driven and fully automatic with selection and order of the splits being optimal.

This model makes biological sense. Leptin levels are associated with BMI^24^, and obesity has frequently been associated with changes in the composition of the gut microbiome. Interestingly, changes in leptin levels have also been associated with changes in the composition of CD4 + T cells. Increased leptin correlates with an increased Th1: Th2 ratio^26,27^, which may explain why we observe IFNG-related variation in the microbiome^4^ on a high-leptin background. By contrast, low leptin correlates with a decreased Th1:Th2 ratio, which may in turn explain why we observe IL4-related variation in the microbiome^28^ on a low-leptin background. The presence of IL23 partitioning in our model potentially indicates Th17 related variation in the gut microbiome^29^ may also be observed on a low leptin background. Our results therefore support an established relationship between obesity and the microbiome and, furthermore, allow us to generate novel hypotheses about leptin-dependent detection of CD4 +T cell interaction with the microbiome.

### Fitting DM-RPart trees

Splitting the 128 microbiome samples into five subsets based on Leptin, IFNG, IL4, and IL23 is a two-step process. In the first step, microbiome samples are recursively split into two groups based on a covariate and cut point such that samples above or below that cut point are more homogeneous. In this example, the parent node (i.e., the set of all 128 samples) is split into two *children nodes* based on the subjects Leptin value. Finding the cut point is described in the technical paper, but in brief, all cut points over all covariates (i.e., IL-17F, IL-17A, IL-21, IL-22, IL-23, IL-12p40, Eotaxin, IL-4, IL-13, IFNG, Leptin, and TGFB) are tested. The covariate/cut point that splits the samples into two groups is selected and entered into the tree (Fig. 1A) that results in the microbiome samples in the two children node being more homogeneous compared to when the samples are combined in the parent node. This splitting step is repeated on the microbiome samples in each child node (i.e., recursively applied) until a small number of samples, say 5, remain in a node, or the taxa counts are identical in all the samples.

The second step of the DM-RPart algorithm is to find the *right-sized tree* after the first step of fitting a *full tree* partitioned down to a small number of samples within each terminal node. To avoid overfitting the data, the *full tree* is pruned back to a smaller *right-sized tree* by cross-validation and cost-complexity pruning. Cross-validation error is defined as the total squared Euclidean distance between validation data to the mean taxa of each terminal node for a given subtree which is known as the PRESS statistic (see details in Methods section) which becomes smaller as the partition finds homogenous subgroups (see Breiman *et al*., Chpt. 11, Section 5)^7^. The cross-validation results for the Leptin/IFNG/IL4/IL23 tree is shown in Table 1. Column one is the number of terminal nodes or complexity of the subtrees, column two is the cost complexity parameter α for pruning, column three the number of splits in the tree, the fourth column the difference in error between nested trees (rows), and the last column is the mean of 100 times 10-fold cross-validation error for selecting the best size tree. The *right-sized tree* is selected as smallest mean cross-validation level which is five on the full iHMP data.

**Table 1:** The complexity table of recursive partitioning on full iHMP data.

Tree Complexity	Cost Complexity (α)	Number of Splits	Relative Error	Cross-validation Error
1	0.0083	0	1.000	0.10704
2	0.0076	1	0.992	0.10893
3	0.0073	2	0.984	0.10792
5	0.0024	4	0.970	0.10508
6	0.0021	5	0.967	0.10827
8	0.0016	7	0.963	0.10903
9	0.0013	8	0.961	0.10948
10	0.0012	9	0.960	0.10928
11	0.0006	10	0.959	0.10907
12	0.0000	11	0.958	0.10932

Averaged cross-validation measure of the best tree of 100 iterations. Each cell shows the average measure of certain combination of the simulation parameters.

### DM-RPart application to Insulin sensitivity data

We subsequently used DM-RPart to examine whether insulin sensitivity may influence host-microbiome interactions. We combined samples for 27 insulin resistant (IR) and 64 insulin sensitive (IS) based on steady state plasma glucose (SSPG) measurements. The same variables as above plus a categorical variable indicating if the sample was from an IR or IS subject were included. Notably, DM-RPart returned no significant tree when partitioning microbiomes from both IR and IS individuals which are shown in Table 2 where the smallest mean cross-validation error happened in the root node (Tree Complexity 1) which means no split on the insulin resistant/sensitivity data.

**Table 2:** The complexity table of recursive partitioning on insulin sensitivity data.

Tree Complexity	Cost Complexity (α)	Number of Splits	Relative Error	Cross-validation Error
1	0.01113	0	1.0000	0.08750
2	0.00622	1	0.9889	0.09051
3	0.00583	2	0.9826	0.09077
5	0.00551	4	0.9710	0.09060
6	0.00353	5	0.9655	0.09045
7	0.00291	6	0.9620	0.09040
8	0.00223	7	0.9591	0.09051
9	0.00081	8	0.9568	0.09138
10	0.00048	9	0.9560	0.09196
11	0.00001	10	0.9555	0.09227
12	0.00000	11	0.9555	0.09230

Averaged misclassification error rate of the best tree of 100 iterations. The number in each cell is expressed as a percentage.

### Application of DM-RPart to simulation data

In this simulation we assess the performance of DM-RPart under a range of sample size (number of microbiome samples), difference between DM parameters ($\pi$ and $\theta$), and normally distributed covariates (Table 3**)**. For each simulation three microbiome datasets were generated using the Dirichlet-multinomial parameters $(\hat{{\rm{\pi }}},\hat{\theta })$ from the HMP saliva, throat, and tongue body sites. Each body site was associated with a randomly generated normal covariate with mean −1, 0, and 1 for saliva, throat, and tongue, respectively. Three standard deviations were used to allow differing levels of overlap between the groups. The number of reads for each sample is 50,000. The datasets were generated under Dirichlet-Multinomial distribution using the function *Dirichlet.multinomial* in the R package *HMP*.

**Table 3:** Summary of parameters in simulation studies.

Parameters	Value
Sample size per body site	40, 80, 120
Expected taxa abundances per body site	${{\rm{\pi }}}_{1}\,from\,saliva$
	$${{\rm{\pi }}}_{2}\,from\,throat$$
	$${{\rm{\pi }}}_{3}from\,tongue$$
Dispersion parameter	0.08, 0.2, 0.6
Mean of covariate	Mean of G1 = −1, G2 = 0, G3 = 1
Standard deviation of covariate	0.2, 0.35, 0.5

Averaged misclassification error rate of the validation data of 100 iterations. The number in each cell is expressed as a percentage.

The statistical assessment of DM-RPart is defined as the average of mean squared error in each terminal node for the *right-sized* pruned trees. The mean squared error is based on the distance of the samples in the node from the node mean (π) with the smaller distance indicates better fit of the data. Note that in the simulation, the group membership was never used for fitting the tree. Table 4 shows the mean results for 1000 iterations. As expected, the distance goes up as the amount of variability among microbiome samples increase (**θ** increases from 0.08 to 0.6), as the sample size decreases (N from 360 to 120), and as the covariate standard deviation increases (SD from 0.2 to 0.5).

**Table 4:** Averaged of mean squared error (E-03) of the best tree.

Total sample size	SD = 0.2			SD = 0.35			SD = 0.5		
	θ			θ			θ		
	0.08	0.2	0.6	0.08	0.2	0.6	0.08	0.2	0.6
120	1.24	3.18	11.23	1.32	3.22	12.64	1.59	3.62	14.26
240	1.08	2.98	9.65	1.12	3.1	10.52	1.35	3.33	11.91
360	0.97	2.69	9.23	1	2.93	9.78	1.25	3.25	10.23

The complexity table of Gibbs-RPart on Parkinson’s disease data. The red row (row 4) indicates the best size tree with 6 terminal nodes.

## Discussion

The Dirichlet-multinomial distribution was introduced previously for formal hypothesis testing in microbiome research^15^ and is extended here for microbiome regression. This paper introduces a novel approach of applying recursive partitioning to find covariates that separate and explain differences in microbiome taxa count data. The utility of this approach lies in discovering covariates associated with the microbiome taxa counts, and for forming hypotheses for future testing and patient stratification in clinical trials.

DM-RPart uses recursive partitioning to regress microbiome compositional data onto covariates. Microbiome taxa count data is compositional since a fixed number of sequence reads are sampled and an increase in one taxa must be accounted for by a decrease in one or more other taxa to ensure that the sum of the proportions always adds to 1^30^. The Dirichlet-multinomial distribution analyzes microbiome compositional data using taxa counts avoiding the need to rarefy which is known to cause loss of information^31^, the reduction of multivariate taxa counts into a single number diversity measure which is difficult to interpret^32^, and the fallacy that systems biology can be learned by univariate statistics through one-taxa-at-a-time analyses^33^.

Any covariates can be used for fitting trees such as treatment or disease group, age, gender, or metabolites. In the first example we focused on cytokines in order to develop hypotheses about microbiome/cytokine associations. In the second example we were interested in whether insulin resistant versus insulin sensitive patients had different microbiomes.

The selection of the covariates is not limited, though we encourage covariate sets be selected that can be interpreted biologically versus adding hundreds or thousands of covariates that will be difficult to understand and result in spurious results due to the high dimensionality of the data^34,35^.

Two known limitations of recursive partitioning are the tendency to overfit the data, and binarizing of continuous variables at each split of the tree into two levels. We address the overfitting problem using a cross-validation cost-complexity pruning algorithm. The issue of splitting continuous variables (binarizing) has been argued as a reason to avoid recursive partitioning algorithms. Nevertheless, RP is a commonly used exploratory analysis tool in the statistics community, and DM-RPart clearly provides insights into the relationship between covariates and the microbiome that cannot be quantified by other approaches. As an exploratory tool it also needs to be kept in mind that the results should be used to advise on the hypothesis testing and design of future studies. We believe the problem of binarizing covariates is outweighed by the insight this model provides.

Other methods are used in microbiome research to associate microbiome data with covariates such as PERMANOVA^36^ or multiple co-inertia analysis (MCIA)^37^. However, these methods differ from DM-RPart in terms of the type of outcome (e.g., PERMANOVA regresses pairwise differences of diversity onto covariates) or how the results are presented (e.g., MCIA presents ordination plots based on taxa counts and covariates). Because these methods and DM-RPart are using different data and approaches a benchmarking comparison was not felt appropriate.

Models fit using the DM-RPart function in R can be used to predict the terminal node assignment and microbiome for new data with the same covariates. The ability to predict in R can be used to validate the models as well and provide independent test data estimates of accuracy.

## Methods

### Introduction to Dirichlet multinomial distribution

The Dirichlet Multinomial (DM) probability distribution on the set of microbiome taxa count samples is defined as:

$$P({{\boldsymbol{{\rm X}}}}_{i}=x\,|{\boldsymbol{\pi }},\,\theta )=(\frac{{{\rm{{\rm N}}}}_{i\cdot }!}{{x}_{i1}!\cdots \,{x}_{iJ}!})\frac{{\prod }_{j=1}^{J}{\prod }_{r=1}^{{x}_{ij}}\{{\pi }_{j}(1-\theta )+(r-1)\theta \}}{{\prod }_{r=1}^{{N}_{i\cdot }}(1-\theta )+(r-1)\theta },$$

where $${{\rm{X}}}_{i}=({x}_{i1},\,{x}_{i2},\cdots ,{x}_{ij})$$ is a microbiome sample with $${x}_{ij}$$ the number of reads of taxa $$j$$ found in sample $$i$$, $$j=1,\ldots \,,J$$ unique taxa and $$i=1,\ldots ,N$$ microbiome samples. The parameter $${\boldsymbol{\pi }}=\{{\pi }_{1},{\pi }_{2},\cdots ,\,{\pi }_{J}\},$$$$0\le \,{\pi }_{j}\le 1,\,\mathop{\sum }\limits_{j=1}^{J}{\pi }_{j}=1$$ is a vector of the expected taxa proportions, and the parameter $$\theta \ge 0$$ is the overdispersion which is a measure of the between sample variability. The interpretation of $$P({x}_{i};\,{\boldsymbol{\pi }},\,\theta )$$ is the probability of observing a sample $${X}_{i}$$ in a group given the parameters $$({\boldsymbol{\pi }},\theta )$$. In practice, $$({\boldsymbol{\pi }},\theta )$$ are unknown so can be estimated using either the maximum likelihood estimation (MLE) or the method of moments (MoM). In this paper, MoM is chosen since MLE is time consuming. Mathematical details on the Dirichlet-multinomial and MoM calculations are presented in La Rosa *et al*.^15^.

### Log-likelihood ratio (LLR) statistic

In DM-RPart, we use the likelihood ratio statistic from the DM model to partition the covariate space into non-overlapping homogeneous subregions. Let $L(\hat{{\rm{\pi }}},\hat{\theta };{x}_{1},{x}_{2},\ldots ,{x}_{N})$be the *likelihood function* of the observed microbiome samples ${x}_{1},{x}_{2},\ldots ,{x}_{N}\in X$ given the method of moments (MoM) parameter estimates $(\hat{{\rm{\pi }}},\hat{\theta })$. The larger the likelihood the more confidence that the data were generated from the model with parameters $(\hat{{\rm{\pi }}},\hat{\theta })$. The likelihood function is calculated as the product of the probabilities of each observed sample,

$$L(\hat{{\rm{\pi }}},\hat{\theta };{x}_{1},{x}_{2},\ldots ,{x}_{N})={\prod }^{}P({x}_{i};\,\hat{{\rm{\pi }}},\hat{\theta }).$$

In classical statistics a *likelihood ratio test* is routinely used for testing alternative models, and was used to test if two sets of microbiome samples come from the same DM model or from different DM models^15^. Formally, the likelihood ratio test (LRT) of the hypothesis, ${H}_{0}:({{\boldsymbol{\pi }}}_{1},\,{\theta }_{1})=({{\boldsymbol{\pi }}}_{2},\,{\theta }_{2})$ is performed by calculating three likelihood functions: ${L}_{1}={L}_{1}({\hat{{\rm{\pi }}}}_{1},{\hat{\theta }}_{1};{x}_{1},{x}_{2},\ldots \in Group\,1)$ where the samples come from one group, say treated, ${L}_{2}={L}_{2}({\hat{{\rm{\pi }}}}_{2},{\hat{\theta }}_{2};{x}_{1},{x}_{2},\ldots \in Group\,2)$ where the samples come from a different group, say controls, and ${L}_{0}={L}_{0}({\hat{{\rm{\pi }}}}_{0},{\hat{\theta }}_{0};{x}_{1},{x}_{2},\ldots \in Group\,1\,and\,2)$ where the samples from the two groups are assumed to come from the same group, such as when the treatment is ineffective and the same as the control. For statistical hypothesis testing the likelihood ratio statistic $LR=\frac{{L}_{0}}{{L}_{1}\times {L}_{2}}$ has well-known statistical properties and is the basis that used to calculate a P value for hypothesis testing.

Instead of hypothesis testing to calculate a P value, in the DM-RPart algorithm LR is used as a measure of how similar and dissimilar subsets of microbiome samples are to each other. In practice for computational accuracy, the log-likelihood $LL({\boldsymbol{\pi }},\,\theta ;{x}_{1},{x}_{2},\ldots ,{x}_{N})={\sum }^{}\,\log (P({x}_{i};\,{\boldsymbol{\pi }},\,\theta ))$ is used in place of the likelihood function but behaves mathematically the same way. The LR is therefore replaced with the log-likelihood ratio (LLR),

$$LLR=L{L}_{0}({{\boldsymbol{\pi }}}_{0},\,{\theta }_{0};{x}_{1},{x}_{2},\ldots ,{x}_{{N}_{1}+{N}_{2}})-(L{L}_{1}({{\boldsymbol{\pi }}}_{1},\,{\theta }_{1};{x}_{1},{x}_{2},\ldots ,{x}_{{N}_{1}})+L{L}_{2}({{\boldsymbol{\pi }}}_{2},\,{\theta }_{2};{x}_{1},{x}_{2},\ldots ,{x}_{{N}_{2}})),$$

with $LLR\approx 0$ indicating the two groups are similar. The further LLR is from 0 the stronger the evidence that the samples come from two groups. In DM-RPart the loglikelihood functions for the microbiome samples combined in a single group $L{L}_{0}$ and split into two subgroups $L{L}_{1},L{L}_{2}$ are used to decide how strong the evidence that the samples should be partitioned into two subgroups (see below for details).

### Introduction to recursive partitioning

**Figure 2:**
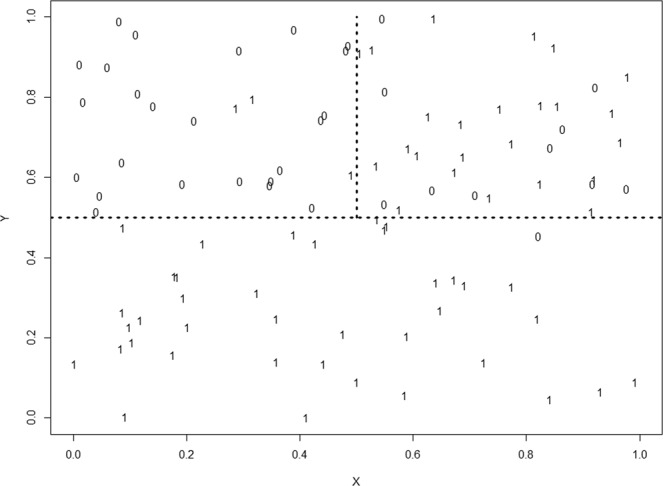
Simulated data to illustrate classical recursive partitioning.

The general framework of recursive partitioning for a binary (Yes/No) outcome is introduced to illustrate the method. A dataset with N = 100 random observations was generated with two predictor (independent) variables X and Y simulated from a Uniform distribution between 0 and 1, and a binary outcome (dependent) variable *Z* = 1 with probability = 0.95 if Y ≤ 0.5, probability = 0.05 if Y > 0.5, X ≤ 0.5, and probability = 0.70 if Y > 0.5, X > 0.5. Otherwise *Z* = 0. The simulated data is shown Fig. 2 along with the boundaries partitioning the covariate space into non-overlapping subregions with different probabilities of Z = 1.

The goal of recursive partitioning is to automatically find boundaries such as those in Fig. 2 over many covariates. The algorithm does this by calculating how well every possible split along X and Y, defined as the midpoints between ordered values improves the homogeneity of the data in the two subsets relative to the unsplit set. Each possible split is scored according to a defined measure of homogeneity, which for binary outcomes is often the Gini diversity index which is minimized if all the outcomes in a subset are the same. In practice the first and last few cut points would not be tested to ensure a minimum, say 5, samples are in each subgroup. After each cut point along X is scored, the same procedure is applied to the other covariates. After all possible splits over all covariates are scored, the split with the *best* score is selected and the data in a (*parent*) node are partitioned into two non-overlapping (*child*) nodes.

The algorithm proceeds recursively by repeating this step on each child node until all the Z (outcome) variables within a node have the same value, or there are less than some minimal number of observations need for splitting, say 5. The order of the splits defines a *tree* structure with the top node containing all the data, and the *terminal nodes* containing subregions of the covariate space defined by splits along the covariates.

**Figure 3:**
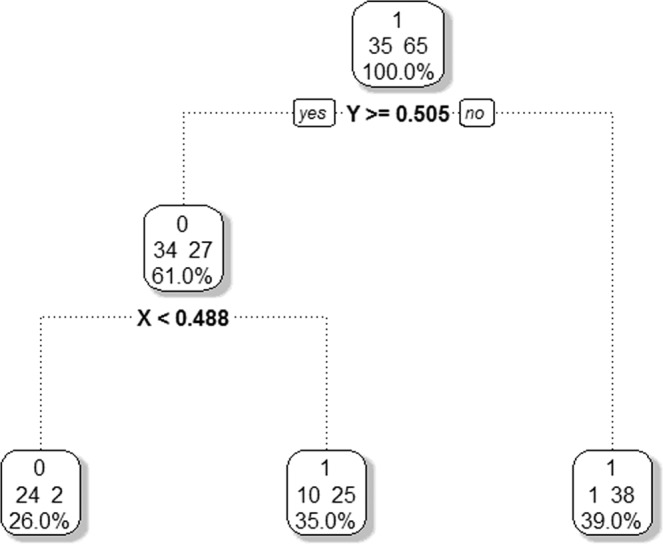
Optimal recursive partitioning tree fit to the simulated data in Fig. 2.

For the simulated data in Fig. 2, 65 samples belong to class 1 and 35 to class 0. Using a majority rule, the data in the *parent* node would be classified as 1 with a misclassification rate of 35% (i.e., the 35 class 0 samples incorrectly classified as class 1). The first split occurs at $Y\ge 0.505$ sending 61 of the 100 samples to the left *child* node, and $Y < 0.505$ sending the remaining 39 samples to the right *child* node. The samples in the right *child* node were not further split with high homogeneity of 38 class 1 samples and 1 class 0 sample for a misclassification rate of 1 out of 39, or 2.5%. The samples in the left *child* node were classified by majority rule as class 0 since 34 out of 61 were class 0, incorrectly classifying the 27 class 1 samples as class 0 samples, a 44.3% misclassification rate. This node was further split along $X < 0.488$ to the left *child* node, and $X\ge 0.488$ to the right *child* node, with misclassification rates of 2 out of 26 (7.7%) and 10 out of 25 (40.0%), respectively (Fig. 3). While the misclassification rate of this last node is high, the overall misclassification rate is 2 class 1 samples misclassified as 0, and 10 + 1 = 13 class 0 samples classified as 1, for a total misclassification rate of 13%, a large improvement over the 35% misclassification from not partitioning the data.

As an exploratory data analysis method^38^, additional attractions of recursive partitioning models are the intuitiveness of the decisions rules defined by the order of the splits down any path, and the ability to fit many variables in the model which otherwise could not be easily displayed visually by other graphical methods.

### Cost complexity and pruning

**Figure 4:**
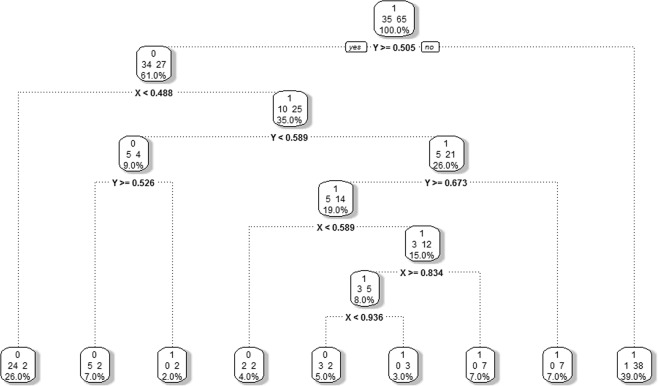
Unpruned full recursive partitioning tree fit to the simulated data in Fig. 2.

To determine the *right-sized tree* such as shown in Fig. 3, the recursive partitioning algorithm fits a full tree, ${T}_{Full}$ until a user-defined stopping criterion such as a minimum number of samples is reached, or a node is found with all outcomes being identical. In the example the full tree shown in Fig. 4 has 9 terminal nodes. To avoid overfitting the data this tree is pruned back using a *cost-complexity parameter* to eliminate branches that balances predictive accuracy with the size (complexity) of the tree^16^. Let ${T}_{Full}$ denote the full tree with 9 terminal nodes from 8 splits (for this example), and $T$ denote a pruned subtree of ${T}_{Full}$ obtained by cutting off branches and collapsing samples in *children* nodes into a higher *parent* node. The complexity of any tree, $\,|T|$, is the number of nodes in $T$, where $\,1\le |T|\le 9$ with $|{T}_{Full}|=9$ and the root node (no splits) $|{T}_{Root}|=1$. Other trees obtained from pruning ${T}_{Full}$ will have complexity ranging between these two extremes.

The cost complexity for any subtree $T$ is

$${C}_{\alpha }(T)=\mathop{\sum }\limits_{n=1}^{|T|}{S}_{n}(T)+\alpha |T|,$$

where $\alpha \ge 0$ is the cost complexity parameter (CP), $|T|$ is the complexity of the tree, and $\mathop{\sum }\limits_{n=1}^{|T|}{S}_{n}(T)$ is the sum over all terminal nodes in $T$ of a measure of homogeneity, which in the case of binary outcomes is usually Gini diversity. The $\alpha$ for each subtree is calculated as the differences of the relative errors of two nested trees, ${T}_{i}\subset {T}_{j}$, where ${T}_{i}$ is obtained by pruning ${T}_{j}$. $\alpha$ is a tuning parameter that balances the tree size $|T|$ and goodness of fit to the data $\,\mathop{\sum }\limits_{n=1}^{|T|}{S}_{n}(T)$.

For a categorical response, the relative error is calculated based on the misclassification rate for each split, with $\alpha$ calculated as the difference of relative error divided by the reduction in complexity. For example, for the tree $\,|{T}_{1}|=3$, $\alpha =\frac{0.371-0.286}{5-3}=0.043$. The optimal $\alpha$ has the smallest cross-validation estimate of error rate. In the example $\alpha =0.043$ has the smallest estimated error rate of 0.429 giving the size of the optimal tree as 2 splits and 3 terminal nodes (Table 5 and Fig. 2). Because pruning uses a nested algorithm there exists only one pruned tree with 2 splits and 3 terminal nodes so a unique tree is obtained.

**Table 5:** The complexity table of recursive partitioning on simulated data.

Tree Complexity	Cost Complexity (${\boldsymbol{\alpha }}$)	Number of Splits	Relative Error	Cross-validation Error
$|{T}_{0}|=1$	0.314	0	1.000	1.000
$|{T}_{1}|=3$	0.043	2	0.371	0.429
$|{T}_{2}|=5$	0.007	4	0.286	0.514
$|{T}_{{Full}}|=9$	0.000	8	0.257	0.743

Pairwise p-value between terminal nodes. The diagonal of the table is one because the pairwise p-value of the terminal node itself is one.

An alternative method of selecting the right size tree is the 1 SE rule. Sometimes the cross-validation errors rapidly decrease for the first splits, followed by a long flat tail with small random fluctuations. When the position of the minimum in the flat tail is unstable, the 1 SE rule provides the simplest tree whose accuracy is comparable to the tree with the smallest cross-validation estimate of error rate. The 1 SE rule is implemented as follows: First define the tree ${T}_{k0}$ with the smallest cross-validation error $R({T}_{k0})$. Second, the tree ${T}_{k1}$ is selected, where ${T}_{k1}$ is the simplest tree such that $R({T}_{k1})\le R({T}_{k0})+SE(R({T}_{k0}))$. Researches could choose either of the smallest cross-validation estimate of error rate or the 1 SE rule to select the right size tree.

### Microbiome recursive partitioning

We presented recursive partitioning using a simple example with a binary outcome to illustrate the method and introduce the mathematics. This method can be used with categorical or continuous outcome variables, and has been extended to more complex models such as linear regression^39^ and genetic epidemiology^10^. Most standard statistical packages now contain versions of recursive partitioning.

In this section, microbiome recursive partitioning is presented that extends classical recursive partitioning to microbiome taxa counts as the outcome variable with splitting based on the *Dirichlet-multinomial log-likelihood ratio* (LLR) and *cross-validation cost-complexity pruning* to avoid overfitting. The goal is to find partitions of the covariate space by recursive partitioning so microbiome samples within terminal nodes are more similar to each other than they are to microbiome samples in other terminal nodes.

Consider a set of taxa count data on *N* ${\rm{subjects}}\,\{{x}_{1},{x}_{2},\ldots ,{x}_{J}\}$. For each subject, a set of *m* covariates $\{{c}_{1},{c}_{2},\ldots ,{c}_{m}\}$ have been measured (e.g., age, gender). Note that recursive partitioning can accommodate missing covariates. Consider a *parent* node A. For covariate ${c}_{m}$, let $c{p}_{{c}_{m}}$be a cut point defined by the midpoints between adjacent values for ${c}_{m}$, such that microbiome of subjects with ${c}_{m}\le c{p}_{{c}_{m}}$ go into the left *child* node, and all others into the right *child node*. As in standard recursive partitioning, the process is repeated for all cut points and covariates, with each split of a parent node scored by the loglikelihood ratio $LLR=L{L}_{ParentNode}-(L{L}_{LeftChildNode}+L{L}_{RightChildNode})$. For each *parent* node, the split with the largest absolute value of $LLR\,$is selected as the split for that node. This process is repeated recursively being applied to each *child* node to fit ${T}_{Full}$. This parameter is specified by the user.

The cost complexity in DM-RP for any subtree $T$ is

$${C}_{\alpha }(T)=\mathop{\sum }\limits_{n=1}^{|T|}{S}_{n}(T)+\alpha |T|$$

where $\mathop{\sum }\limits_{n=1}^{|T|}{S}_{n}(T)$ is the sum of the *LL* over all terminal nodes in the subtree $T$ and $\alpha$ adjusts for the size of the tree. When $\alpha =0$, the largest tree is selected, because $\alpha |T|$ part is dropped. As $\alpha \to \infty$, the root tree will be selected since larger trees result in larger cost-complexity values. For a given $\alpha$, a subtree $\,T(\alpha )$ which minimizes $\,{C}_{\alpha }(T(\alpha ))={\min }_{T\le {T}_{FULL}}\mathop{\sum }\limits_{n=1}^{|T(\alpha )|}{S}_{n}(T)$ can be found because there are finite number of subtrees of ${T}_{FULL}$.

A 10-fold cross-validation method for selecting the right sized tree is developed. In any node we can calculate the MOM estimate of the Dirichlet-multinomial parameter $(\hat{{\rm{\pi }}})$ as the average taxa proportions of taxa samples contained in that node. For a new taxa sample, it will fall into a node based on its covariates and the splitting rules defined by the tree. The Euclidean distance between the sample’s taxa frequency and $\hat{{\rm{\pi }}}$ is calculated. The mean of the squared Euclidean distance of all the new test samples to the $\hat{{\rm{\pi }}}$ of the terminal nodes they fall into is calculated as a measure of fit and used for cross-validation pruning. The procedure is implemented as follows:

1. Fit ${T}_{Full}$, and calculate all cost complexity parameters $\alpha$
2. Randomly partition the data into 10 equal sized subsets.
3. For each subset $i\,(i=1,2,\ldots ,10):$
  - Set the i^th^ subset (D_i_) as the validation dataset
  - Fit the ${T}_{Full}\,$from step 1 to the remaining 90% of the data, called ${T}_{Full90 \% }$
  - Obtain the pruned trees of ${T}_{Full90 \% }$ at each $\alpha$ level from step 1 and calculate MOM $\hat{{\rm{\pi }}}$ for each terminal node of each pruned tree, called ${\hat{{\rm{\pi }}}}_{\alpha 90 \% }$
  - Compute the predicted class for all observations in D_i_ at each $\alpha$ level
  - For each pruned tree, calculate the cross-validation error as the mean of squared Euclidean distances from D_i_ to ${\hat{{\rm{\pi }}}}_{\alpha 90 \% }$
4. Repeat step 3 for all $i\,(i=1,2,\ldots ,10$).
5. Claculated the averaged cross-validation error at each $\alpha$ level of the 10 iterations in step 4.
6. Repeat step 2–5 $M\,(M=1,2,\ldots$) 100+ times to obtain the consensus result
7. The subtree with the smallest averaged cross-validation error of step 6 or follows the 1 SE rule is selected as the right sized tree

### Statements of ethics approval

(I). Participants provided informed written consent for the study under a research study protocol #23602 approved by the Stanford University Institutional Review Board. (II). All research was performed in accordance with relevant guidelines and regulations. Informed consent was obtained from all participants and/or their legal guardians.

## Data Availability

DM-RPart is available in the R HMP package as open source code through CRAN. In addition, the data and code to generate Fig. 1 are included as a vignette and can be used to reproduce our results and as a template for running other datasets.
